# Nosological Observations

**Published:** 1818-06

**Authors:** Thomas Parkinson


*For the London Medical and Physical Journal.*

### *Nosological Observations*; by THOMAS PARKINSON, M.D.

I. NOSOLOGY requires two distinct Nomenclatures, consisting of,

1. CLETICS,* or appropriate names for diseases, fully representing the GENUS of the solid diseased, and the precise nature of the affection.
2. PHANERICS,† or appropriate appellations for Congresses of Evidences, or Symptoms, by which diseases are distinguished.

CLETICS refer chiefly to the Anatomical Table,—as MYELEUTHĪTIS HÆMEUTHYTŌDES, from Μυελος, the substance of the brain; and ευθυς, straight; signifying inflammation of some part of the nervous system; and its quality and precise GENUS is declared by the GENERIC adjective HÆMEUTHYTŌDES, from Αιμα, blood; signifying that it is the first GENUS of the first CLASS of solids which is inflamed:—that is, either some immediate Organ of Sense, or the Brain within the Head, an Association of Brain with Muscle.

PHANERICS refer chiefly to the Physiological Table,—as ÆSTHESĪTIS; signifying morbid excess of function in an Organ of Sense; that is, from Αισθησις, Sense; Perception being the functions of the Organs of Sense; and the elaboration of Perceptions into Mind, the function of the Brain: each of these faculties consists of the *two essential Attributes of Animal Life*—Sensation and Motion; and they are the inevitable consequences of the GENUS of solid MYELEUTHYTŌNA HÆMEUTHYTŌDES. Excess of function, then, in an Organ of Sense, or in the Brain, must be the necessary consequence of Inflammation; and, ÆSTHESĪTIS is a term which comprehends all the distinguishing symptoms of the disease, as relates to its identity; and, its history will be complete, by the addition of its adjective of *Species*,—as Æsthesītis Encephalica, Æsthesītis Ophthalmica, Æsthesītis Stomatica, Æsthesītis Myceterica, Æsthesītis Otonica, Æsthesītis Exoterica. And, let it be remembered, that Æsthesitis must always be present, as an evidence, in Myeleuthitis Hæmeuthytōdes, but cannot have existence without it.

MYELEUTHĪTIS ENYPHANTODES; Ενυφαινω, to intertex; signifying inflammation of an association of Brain with Membrane,—the Nervous Cord, whose only function is to carry Perceptions unchanged to the Brain, and the commands of the Brain to the moving Solid. The Phaneric term, comprehending all the symptoms by which this disease is distinguished, is ÆSTHEPHORĪTIS, from Αισθησις, and Φορεω, carriers of Sense; implying excess of function of the Nervous Cord, which, if proceeding from an organ of Sense to the Brain, will be known by inordinate and incorrect transmissions of Perceptions to the Brain, consequently incorrect judgment of things; or, if proceeding from the Brain to the voluntary Muscles, it will be known by excessive, inordinate, incorrect transmissions of the commands of the Brain to such Muscles: consequently, ungovernable, involuntary, muscular action—Convulsion, Trismus, Tetanus, &c. And, let it be remembered, that ÆSTHEPHORITIS is the necessary consequence, and must always be present, as an evidence, in Myeleuthītis Enyphantodes, but cannot have existence without it.

* Κλητικος; vocatus; denominated.

† Φανερος; manifestus; evinced.

The same concordance of the Cletics and Phanerics will be manifested in my Nosological Tables, which I have just finished, in all the classes and genera of diseases; exhibiting an interesting and luminous view of the Identities of diseases, and the characteristic symptoms by which they are distinguished.

But diseases must not be denominated from their Symptoms, or from their Causes, but from their Identities.

*Quere* 1.—If *A* commits murder, and *B* is the evidence who convicts him; is the crime to be denominated *B*?

*Quere* 2.—If *A* commits murder, and *B* is the evidence who convicts him; is the crime to be denominated *A*?

Symptoms are but Evidences, by which we know that disease exists, and distinguish one disease from another; and they must not be allowed any other power.

I shall take the liberty of putting the following questions:

1. Are not the greater number of diseases, at present, denominated from their Symptoms?
2. Ought they to be so denominated?
3. Cannot diseases be denominated from their Identities?
4. Ought not they to be so denominated?
5. Should we not correctly discriminate between idiopathic and symptomatic affections, by giving them names representative of their intrinsic properties?
6. And should we not rescue Nosology from that opprobrious confusion which infests it, and renders it unworthy of the name of System; fix it upon a solid foundation, and bring it under laws equally inflexible and just?

I shall conclude this paper with a few more examples of Cletics and Phanerics.

Aneurisma; *ανευρυνω*, to dilate much. This term is indeterminate; it points only to the quality of the disease—dilatation, which may be of one solid or of another.

An Artery, as it is called, the subject of the disease before us, is, anatomically considered, Tubulated Elastic Ligament; and, physiologically, Tubular Reaction: the former is denominated Helasmata Siphonodes, from Ελασμα, laminated, and Σιφων, a tube—tubulated laminated fibre: the latter is denominated Siphapokinēsis, from Σιφων απο κινεω, tubular reaction.

To raise the true image of this disease in the mind, I would denominate Aneurisma Platynēsis helasmīca siphonōdes; dilatation of tubulated elastic ligament;—this should stand as Cletic: and the Phaneric, comprehending the Symptoms by which it is to be distinguished from other diseases, is Asiphapokinēsis, a priv., σιφων, a tube; αποκινεω, to react—defective reaction of tubulated elastic ligament.

Rhéumatismus, *ρευματιζω*, to flow down. This term has no relation to inflammation of muscle. The Cletic, expressive of the identity of the disease, is Hæmeuthītis, *αιμα ευθυς*, inflammation of straight fibres with blood—the anatomical characteristic of muscle. And the Phaneric term will fully express the symptoms by which the disease is distinguished; Kinesītis, *κινησις*, action—

excess of function of muscle. And, by adjoining the GENERIC adjective, STEREŌSES, the character of the disease will be explained: as, HÆMEUTHĪTIS STEREŌSES, inflammation of solid muscle; HÆMEUTHĪTIS ANTRŌDES, inflammation of the heart.

ENTERITIS, *εντερα*, the bowels; signifying inflammation of the intestines, is not sufficiently anatomical,—*intestinum*, from *intus*, within, not being a determinate denomination. The disease is fully defined by ENYPHANTĪTIS HÆMEUTHYTŌDES; signifying inflammation of membrane associated with muscle—the anatomical characteristic of the intestinal canal. The PHANERIC corresponds in the denomination,—PARENTHISYSTOLĪTIS, *παρενθεσις*, insertion, or interposition; and, *Συϛελλω*, to contract, the combined function of the association of Enyphanta and Hæmeuthytona; signifying excess of this function.

PERIPNEUMONIA, *περι πνευμων*, about the lungs, is not sufficiently determinate. ENYPHANTĪTIS HELASMATŌDES identifies inflammation of the association of membrane with elastic ligament, the anatomical characteristic of the substance of the lungs, and of the *corpora penis;* and the Species will be distinguished by the adjectives denoting situations—as, ENYPHANTĪTIS HELASMATŌDES Thoracica, inflammation of the lungs; ENYPHANTĪTIS HELASMATŌDES Gonica, inflammation of the *corpora penis.*

The PHANERIC term, PARENTHAPOKINĪTIS, *παρενθεσις αποκινεω*, excessive interposition and reaction, declares the symptoms.—And so on.

This view of Nomenclature shows the necessity of teaching Nosology by CLETICS; and that diseases may be elegantly described by PHANERICS: it will, moreover, prepare such of your readers as may be so inclined, to read my Tables of Medical Nomenclature, which I intend to lay before the public without delay.

*Leicester; April* 10, 1818.

---

